# The Institutional Library

**Published:** 1920-01-03

**Authors:** 


### THE INSTITUTIONAL LIBRARY.

**A Guide to Gynæcology in General Practice.** By COMYNS BERKELEY, M.D., F.R.C.P., and VICTOR BONNEY, M.D., F.R.C.S. (Frowde and Hodder and Stoughton. Second Edition. Price 31s. 6d. net.)

**Diseases of Women.** By T. G. STEVENS, M.D., F.R.C.S. (University of London Press: Hodder and Stoughton. New Edition. Price 20s. net.)

THESE two books on the same subject—namely, gynæcology—and both from Warwick Square, illustrate the different standpoints from which a single science can be studied and expounded. Dr. Stevens is concerned mainly with students of medicine *in statu pupillari*; Drs. Berkeley and Bonney write not at all for this class, but for the qualified man in actual practice. The extraordinary difference in the resulting books represents in some degree an unavoidable but regrettable divorce between theory and practice in this branch of medicine, but is probably also partly attributable to the personal factor of authorship. Dr. Stevens, with considerable justice, insists throughout on the pathological basis of classification: he wants to convince the student of the essential unity of the causes of disease and of the basic principles of treatment in pelvic as in other diseased processes. He wants to speak to the student in terms with which he is familiar, to dwell more on the similarities of pathological change than on the differences due to pelvic anatomy and to the peculiar functions of the (female) pelvic organs. That he succeeds in this attempt no one can deny; but he correspondingly runs the risk of underemphasising the clinical side of the subject. On the whole he has maintained a good balance, bearing in mind that his text-book is essentially one for the unqualified student.

On the question of the prevention and treatment of shock, Dr. Stevens favours prophylactic saline infusion to the extent of five pints in an operation of fifty minutes, and goes on to recommend 15 to 20 ounces every four hours *per rectum* for the developed condition. This is rather too much of a good thing: a patient treated thus might receive eleven or twelve pints of saline in twenty-four hours and would stand some risk of œdema of the lungs. The gum method and blood transfusion are not mentioned. He also places more faith in pituitrin for surgical shock than most surgeons do. In stating his views on anæsthesia, no mention is made of combined spinal and inhalation anæsthesia for Wertheim's and similar operations. Here and there ambiguities of expression are to be found which a student's text-book should especially avoid.

Drs. Berkeley and Bonney, on the other hand, are as clinical and practical as Dr. Stevens is pathological. Their collaboration has long been one of the outstanding features of British medical authorship, and this volume is worthy to set beside the best of their series. A better book on a special subject for the help and guidance of the intelligent general practitioner is hard to recall. Both diagnosis and treatment are most satisfactorily dealt with.

The special chapters on the medico-legal aspects of gynæcology are exceedingly full and valuable: the only possible criticism of them is that the authors do not include in their advice to practitioners any mention of the medical defence societies. The sections on dyspareunia are also worthy of special mention for helpful advice: speaking broadly, the authors agree with Mrs. Stopes' frank discussion of female sexuality, though in one or two points they diverge. To judge from the illustrations as well as the text, the authors do not believe in the usual surgical teaching that abdominal palpation should be done with the flat hand; they prefer the finger-tips. They use the term "diathermy," apparently, for the method of slow coagulation at comparatively low temperatures, not for the superficial burning produced by the high-frequency alternating current. Neither text-book mentions the value of this "cold cautery" method for inoperable pelvic malignant disease, one of the very few omissions of practical therapeutics from the "Guide." The index to Drs. Berkeley and Bonney's book is as exemplary as that to Dr. Stevens' is the reverse.

**Groundwork of Surgery.** By ARTHUR COOKE, M.A., M.B., B.Ch. (Oxon), M.A. (Cantab), F.R.C.S., Surgeon to Addenbrooke's Hospital, Cambridge, and University Teacher in Surgery. (Cambridge: Heffer and Sons, Ltd. Price 7s. 6d. net.)

THIS small volume is intended primarily for students just commencing surgery, and we agree with the author in finding it a common experience that the average man, when he begins clinical work in the wards or out-patient department, is lost in a maze of local lesions, and that the larger systematic textbooks are too complete to give him the help which he immediately needs. With this object in view, the whole subject is set out in extremely simple and straightforward language, and apart from giving valuable aid to the first-year student it will probably find a definite use in bringing before men more advanced in their training some of those simple truths which are so frequently lost sight of both in practice and the examination room. One suggestion might be made, and that is that diagrams and simple line illustrations of some of the practical subjects dealt with, would undoubtedly aid those who experience the common difficulty of fixing in mind textbook matters when given in type alone. Otherwise the chapters and general production are admirable.

**A Public Medical Service.** By David McKail, M.D., and Wm. Jones. (Fabian Society; and George Allen and Unwin. Pp. 72. Price 1st. net.)

THE authors of this pamphlet are strongly in favour of the "nationalisation" of the medical profession: of making all medical men, in other words, direct salaried servants of the State as officers of a State Medical Service. They marshal very ably and cogently the case against an extension of the present Panel system, and advocate its replacement by some other principle whenever the apparently near extension of State medical aid takes place. They proceed to outline a scheme, admittedly but tentative, for providing a complete medical, nursing, and institutional service for the whole community, with especial reference to the cost of it. There is much that is interesting and valuable in their estimates, which work out (for urban areas) at a trifle over twelve shillings per annum per head of population. It must be noted, however, by all who study these estimates, (*a*) that salaries and costs of all kinds are reckoned on a pre-war basis, (*b*) that no provision whatever is included for capital expenditure on new buildings and equipment, (*c*) that all existing institutions and their revenues are to be commandeered, and their value is not included in these estimated costs, and (*d*) that on the question of salaries for doctors, nurses, administrative officers, etc., no provision whatever appears to have been made for pensions or superannuation allowances. From the point of view of finance,

**The Institutional Library**—(*continued*).

the pamphlet therefore is not altogether to be taken as all-inclusive. At the same time, it is a pleasure to acknowledge the thorough-going way in which the framework of a complete organisation has been worked out, and the obvious keenness for improving the health of the nation which throughout inspires the authors. Their sincerity is as clear as daylight, and their suggestions are constructive: accordingly, their sketch of a public medical service deserves careful consideration, and is a real contribution to a very difficult and thorny problem which is certain to come up for solution within the next few years.

**Letters to a Young Man on Love and Health.** By Walter M. Gallichan. (London: Werner Laurie. Pp. 123. Price 4s. 6d. net.)

Now that traditional British Puritanism is breaking down, at least to the extent that some instruction in sex questions is recognised as desirable for adolescents, the need for books which shall guide parents and schoolmasters, or shall be fit to place directly in the hands of those to be instructed, has resulted in a good supply of books written to meet that demand. Some of these are more plainspoken than others: perhaps in one or two cases too plainspoken; others are not quite clear and frank enough, and it is in the latter class that we should feel inclined to place Mr. Gallichan's book. Certainly he is throughout "on the side of the angels": so much so at times as to make his counsel almost too Olympian for this workaday world. He constantly recognises the dangers of pedantry, of platitude, of prosiness, and of preaching; for he frequently begins his chapters with disclaimers of any such intentions. But, in spite of his endeavours, it is just these dangers that he is apt occasionally to fall into. Still, it is easy to see the excellence of his motives, and the devotion he has displayed in attempting what must always be a very difficult literary task. There are a great number of erroneous statements to be found in the text; some of these are of small importance, but others very seriously affect the argument and detract materially from the usefulness of the book.

**Handbook of Skin Diseases.** By Frederick Gardner, M.D., B.Sc., F.R.C.S.E. (Edinburgh: E. and S. Livingstone. Price 6s. net.)

This is a most useful little volume on a subject which is chiefly remarkable for the relatively few medical men who even approximately understand it, and in these pages, which are the direct outcome of the author's lecturing experience, most valuable information will be gleaned rapidly and without the usual strain of textbook study. The various conditions are taken in a somewhat curious order, but inasmuch as the commoner diseases appear first, this classification is probably a great advantage to the beginner, even if it is not scientifically correct. The photographic illustrations are extremely good, and, on the whole, we congratulate the author on producing as useful a guide as we have seen to the early study of skin lesions.

**Letters from Another Battlefield.** (London: Erskine Macdonald. 1s. net.)

This little book was first published in May 1916, the second year of the Great War. It consists in a series of letters written by one who is condemned to fight the ravages of the insidious disease of consumption, to one who is taking his part in the fight against the nation's enemies abroad. The lives of both are lost, and the publisher in his note draws the attention of the public to the fact that as many people in our country die of tuberculosis each year as the number of soldiers who fell during the first year of the war. The book is published as a plea for the recruitment of a strong army "to enter the lists in combat with this horrible enemy which wrecks the lives not of men only, but of women and children."

**Manual of Diseases of Children.** By James Burnet, M.D. Second Edition. (E. & S. Livingstone. 1919. Pp. 416. Price 8s. 6d. net.)

The war has been responsible for the rather long interval between the first and the present issues of this manual of diseases of children; the new edition was nearly ready when the war broke out, and was in consequence held up. Broadly speaking, this elementary textbook presents an excellent outline sketch of its subject. The author, it may be noted, is less dogmatic in some of his opinions than he was formerly, a common pheonomenon as experience lengthens and wisdom ripens. The most important chapters of any book on children's diseases are usually those on feeding. Here it is remarkable that the author sticks to the two-hourly system of feeding for infants during the first month of life, and does not lengthen the interval to three hours until the fourth month. Granting that there are some babies who cannot advantageously be fed three-hourly right from birth, yet there are a great majority who can, and very, very, few indeed that cannot be thus nursed at three or four weeks. His table of average increase of weight in nurslings is rather on the conservative side too; it makes the average gain for the first year exactly thirteen pounds, which is less than most authorities regard as normal. He is very strongly against all dried milks. Altogether a very useful guide for the student and the not too experienced general practitioner.

**Pocket Guide to the First Aid to be Rendered in Cases of Poisoning.** By Col. R. J. Blackham, C.B., C.M.G. (Second Edition. Price 6d. net. Wiles and Son, printers, Colchester.)

The need of knowledge and what to do in cases of poisoning is universally required among all classes, and should be widely circulated in language as simple as possible, so that understanding may be clear and action prompt in event of accident or emergency. Such a pocket guide as that written by Colonel R. J. Blackman has within it useful and necessary information, and should find its way into every pocket and every home. There is still a need to call the poisons in common use by their household name by which they are more familiarly known; also the treatment of these in household language. Condy's Fluid is known to all, but only a privileged minority understand it as permanganate of potash.

**A Medley of Verse** By F. Barber-Wells, M.B. (W. H. & L. Collingridge, 148-9 Aldersgate Street, E.C. Price 2s. 6d. net.)

In the present volume Dr. Barber-Wells has reprinted some of the war verses which were originally published in "The Roll of the Drum." The welcome which that work received will probably be given to its successor, of which the war verses form less than a third. The best in the volume is "His Epitaph," and the general appeal is more to the public at large than to those who have cultivated a critical taste in literature. It is only fair to add that, when the price is considered, the paper and appearance of the book deserve praise.

